# AI-based color vision screening and educational counseling for high school students: An experimental study

**DOI:** 10.1371/journal.pone.0353871

**Published:** 2026-07-22

**Authors:** Dang Vinh, Phan Thi Yen

**Affiliations:** 1 Faculty of Digital Economy and E-Commerce, Vietnam - Korea University of Information and Communication Technology, The University of Danang, Danang, Viet Nam; 2 Department of Research and International Cooperation, University of Foreign Language Studies, The University of Danang, Danang, Viet Nam; Khoy University of Medical Science: Khoy University of Medical Sciences, IRAN, ISLAMIC REPUBLIC OF

## Abstract

Color vision deficiency (CVD) can negatively affect students’ learning experiences and career choices; however, combined screening and educational counseling remain challenging in school settings, particularly in low-resource contexts. Conventional paper-based color vision tests are time-consuming, difficult to standardize, and rarely linked to individualized educational or career guidance. This study aimed to design, implement, and validate an Android-based mobile application that integrates CVD screening with artificial intelligence (AI)–assisted educational and career counseling for high school students.

A cross-sectional study was conducted with 527 high school students in Danang, Viet Nam. The application incorporated a digital Ishihara-based screening module and an AI-assisted counseling component. Screening outcomes were compared with conventional Ishihara testing to evaluate agreement, while students’ perceptions of usability, usefulness, and counseling relevance were assessed using a validated questionnaire. Statistical analyses included descriptive statistics, independent-samples t-tests, reliability analysis using Cronbach’s alpha, and exploratory factor analysis.

Seventeen students (3.23%) were identified as having CVD, including red-green, protan, deutan, and achromatopsia (complete CVD). The mobile application demonstrated high concordance with conventional screening results. No statistically significant differences were observed between students with and without CVD in overall system evaluation scores (p > 0.05). The questionnaire showed good internal consistency (Cronbach’s alpha = 0.857) and a clear multi-factor structure reflecting students’ acceptance of AI-assisted educational and career counseling.

These findings indicate that the proposed mHealth application offers a feasible and scalable approach for school-based CVD screening combined with personalized educational and career guidance. The system may serve as a supportive decision-making tool for students and counselors, while further validation under diverse real-world conditions and across different mobile devices is recommended.

## Introduction

Color vision deficiency is a common visual disorder that affects an individual’s ability to accurately perceive and differentiate colors, particularly along the red-green spectrum. Globally, it is estimated that approximately 8% of males and 0.5% of females have some form of CVD [1]. Among school-aged students, CVD may impair not only the learning of color-dependent subjects such as biology, chemistry, and geography, but also everyday classroom activities that rely on color-coded information [2]. Previous studies have shown that students with CVD encounter difficulties in interpreting maps, graphs, and instructional materials that use color as a primary means of communication, which can negatively influence academic performance and learning confidence [3]. In Viet Nam, Nguyen et al. [4] reported that approximately 2% to 5% of high school students may have CVD; however, a substantial proportion of cases remain undiagnosed, highlighting an ongoing challenge for early identification and educational support.

Traditional methods such as the Ishihara plates, the Farnsworth-Munsell 100 Hue Test, and the D-15 test are widely used for detecting CVD [1]. Among these, the Ishihara test is the most commonly applied in clinical and educational contexts due to its simplicity and rapid administration; however, it has limited capacity to differentiate detailed subtypes and severity levels of CVD [5]. More comprehensive assessments, such as the Farnsworth-Munsell test, can provide more precise diagnostic information but require longer administration time, controlled testing conditions, and trained personnel. These requirements reduce their practicality for large-scale implementation in school settings, particularly when repeated screening and individualized educational counseling are needed. As a result, conventional approaches remain difficult to standardize and scale within routine secondary school screening programs.

Recent advances in information technology and AI have facilitated the development of digital tools for CVD detection, aiming to improve accessibility, objectivity, and efficiency in screening processes [6]. Mobile applications and AI-powered systems have been proposed to automate visual assessment and reduce reliance on manual interpretation. For example, Wang et al. [7] introduced an AI-based screening system that processes visual input data to classify CVD types, while Bitkina et al. [8] applied image processing and machine learning techniques to minimize human error and enhance diagnostic objectivity. Despite these technological advances, most existing systems primarily focus on algorithmic performance and are evaluated in controlled or clinical environments, with limited integration into routine school-based screening programs.

More recently, AI has been applied to support individuals with CVD through digital image processing. Prajapati and Goyal applied machine learning techniques to enhance Ishihara plates and educational images, aiming to improve visual accessibility for learners with red-green CVD. Their findings highlight the growing role of AI in addressing visual accessibility challenges; however, such approaches primarily focus on image enhancement rather than large-scale screening or educational deployment in real-world school settings [9].

Beyond screening technologies, AI has also been increasingly explored as a tool for enhancing educational and career guidance for high school students. AI-assisted counseling systems enable data-driven personalization, facilitate access to career-related information, and help overcome structural limitations of traditional counseling approaches. Evidence from the Vietnamese educational context suggests that students with specific needs, including those with perceptual or visual limitations such as CVD, demonstrate a higher demand for and trust in AI-supported guidance systems. These insights indicate that combining early screening with AI-assisted educational counseling may provide a more inclusive and personalized support framework within school settings [10].

At a broader level, AI has increasingly been applied in educational counseling to personalize learning pathways and support students’ academic and career development [11]. AI-driven counseling systems that combine machine learning, natural language processing, and multi-dimensional data analysis have demonstrated potential in generating personalized career recommendations and improving counseling efficiency [12]. Such approaches are particularly relevant for students with specific needs, including those with visual or perceptual limitations such as CVD. However, in Vietnam, the application of AI in educational counseling remains at an early stage. Initial studies and pilot initiatives suggest considerable potential, yet challenges persist in terms of technological infrastructure, device availability, and staff readiness [13].

Collectively, international and domestic studies indicate growing interest in AI-driven tools for both CVD assessment and educational counseling. While prior research by Khizer et al. [6], Wang et al. [7], and Westman et al. [12] has demonstrated the technical feasibility of AI-based screening and counseling systems, these studies have largely examined detection accuracy or recommendation performance in isolation. Limited attention has been given to the combined implementation of CVD screening and AI-supported educational counseling within real-world secondary school settings, particularly in low- and middle-income contexts. In Vietnam, the work of Nguyen et al. [4] provides important epidemiological insights into CVD prevalence but does not address scalable, technology-enabled screening or counseling models for schools.

In response to these gaps, the present study aimed to develop and evaluate a mobile-based CVD screening application incorporating AI-assisted functionality to support educational counseling for high school students in Danang, Vietnam. Specifically, the study sought to (1) assess the diagnostic accuracy and agreement of the software compared with conventional screening methods, (2) examine its impact on educational and career guidance outcomes, and (3) explore students’ satisfaction and perceptions regarding the usability and relevance of AI-assisted counseling interventions in a real-world school context.

## Materials and methods

### Description of the color vision screening application

The color vision screening application was developed as a mobile-based system for use on the Android platform, with a focus on usability, accessibility, and scalability in real-world educational and community settings. Although the application is designed to be applicable to users across different age groups, the present study evaluated its implementation and effectiveness among high school students. The user interface was developed to be simple and intuitive, ensuring ease of use for both adolescent and adult users.

The screening module is based on a digitized version of the Ishihara color vision test (2000 edition), integrated into the mobile-based application. Test items were presented sequentially on the device screen, and participants were instructed to read the numbers or trace color-defined paths embedded in pseudoisochromatic plates, depending on the test format. Based on participants’ selections, the application evaluated color perception performance and supported the classification of different types of CVD, including red-green, protan, deutan, and achromatopsia. All responses were recorded according to the choices made by the screened individuals and securely stored within the system for subsequent analysis.

**Fig 1:**
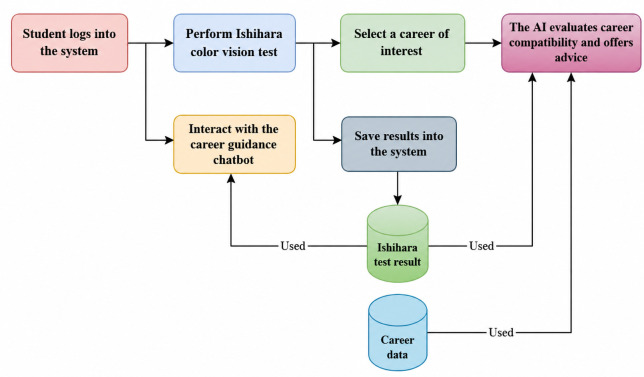
Workflow of the AI-assisted CVD screening and educational and career consulting system.

The system incorporates AI-assisted functionalities with the following components (Fig 1):

1. Image processing and response recognition: Based on users’ selections in the digitized Ishihara test, the system analyzes response patterns and supports the classification of color vision status, including normal vision, red-green CVD, protan, deutan, and achromatopsia.
2. AI-assisted educational and career counseling: Based on screening outcomes and user-selected learning interests, the system generates rule-based educational and career guidance, including suggested subject focus areas, learning strategies, and potential career pathways appropriate to the user’s color vision profile.
3. Automatic data recording and management: The application records users’ selections and screening outcomes and stores them for subsequent statistical analysis and evaluation.

### Participants, sampling, and data collection procedures

The study was conducted with 527 high school students from various schools in Danang, Viet Nam. Participants were recruited using a convenience sampling method to facilitate access across schools while ensuring diversity in gender, age, and academic level. Recruitment and data collection were carried out between June 15, 2024, and May 10, 2025.

Students with known ocular diseases or acute visual conditions unrelated to CVD were excluded from participation. The data collection procedure involved administering the mobile-based screening application, followed by completion of an evaluation questionnaire assessing user experience and perceptions of the AI-assisted counseling system. A subset of participants was additionally assessed using the conventional paper-based Ishihara test to examine agreement between traditional and app-based screening outcomes.

**Fig 2:**
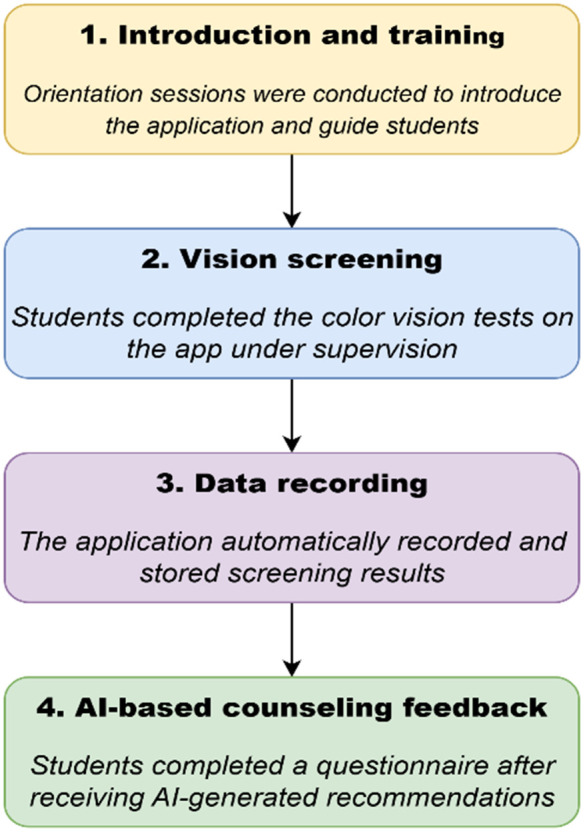
Data collection procedure.

The data collection procedure consisted of the following steps (Fig 2):

### Ethical considerations

This study involving human participants was reviewed and approved by the Biomedical Research Ethics Committee of Danang Hospital, Viet Nam (Approval number: 1299/BVDN-HDYD, issued on June 4, 2024). Written informed consent was obtained from all participants and their legal guardians prior to participation. All procedures were conducted in accordance with institutional guidelines and the principles outlined in the Declaration of Helsinki. All data collected through the mobile application were anonymized prior to analysis and securely stored. Access to digital data was restricted to the research team, and the information was used solely for research purposes.

### Instruments for evaluating AI-driven counseling impact

Students’ perceptions of the application were assessed using a structured questionnaire administered after completion of the screening process. All items were measured using a five-point Likert scale (1 = lowest perceived rating, 5 = highest perceived rating). The questionnaire comprised two main sections:

1. *Evaluation of the mobile-based educational and career counseling application:* This section evaluated the AI-assisted educational and career counseling component and included items measuring awareness (AW), trust in AI (TR), perceived benefits (BE), barriers and security (BR), system expectations (EX), and willingness to use (RD).
2. *Evaluation of the mobile-based CVD application:* This section assessed the mobile-based CVD screening component, with items measuring overall satisfaction (OS), quality and accuracy (QA), time and convenience (TC), enjoyment and user experience (EE), comparison with traditional methods (CT).

Statistical analyses were performed using SPSS software. Descriptive statistics were used to summarize participant characteristics and screening outcomes. Independent-samples t-tests were conducted to compare overall satisfaction scores between students with and without CVD. Internal consistency of the evaluation instruments was assessed using Cronbach’s alpha.

Exploratory factor analysis was conducted using principal component analysis with Promax rotation to examine the underlying factor structure of the questionnaires. Confirmatory factor analysis was subsequently performed to evaluate model fit. Statistical significance was set at p < 0.05 for all analyses.

## Results

### Color vision screening results via the application

The screening was conducted under real-world school conditions involving 527 high school students. The color vision screening performed using the developed mobile application identified a total of 37 students with potential color vision abnormalities, of whom 17 students were identified as having CVD. This finding is consistent with previous epidemiological evidence indicating a markedly higher prevalence of congenital CVD in males compared with females.

The remaining 20 students were categorized as exhibiting color vision weakness or inconclusive screening results. These cases were not classified as definitive CVD and may require further clinical assessment for confirmation. Accordingly, subsequent analyses focused on the 17 students identified as having confirmed CVD.

A detailed analysis of the types and distribution of CVD among these 17 students is presented in Table 1.

**Table 1:** Frequency of types of color vision deficiency among students.

Types of color vision deficiency	Male		Female	
	Quantity	%	Quantity	%
Total number of students	308	100	219	100
Red-green CVD	6	1.95 ± 1.55	0	0.00 ± 0.00
Protan	7	2.27 ± 1.67	0	0.00 ± 0.00
Deutan	3	0.97 ± 0.56	0	0.00 ± 0.00
Achromatopsia	1	0.32 ± 0.63	0	0.00 ± 0.00
Total number of students with CVD	17	5.52 ± 1.30	0	0.00 ± 0.00

Red-green CVD, including protan and deutan subtypes, accounted for the majority of identified cases. One student was classified as having achromatopsia.

These results indicate that red-green CVD, including protan and deutan subtypes, accounted for the majority of CVD cases identified in the sample. No female students were classified as having CVD, which is consistent with established genetic patterns of congenital CVD.

To validate the screening accuracy of the mobile application, a subsample of students was additionally assessed using the conventional paper-based Ishihara test. The app-based screening results showed agreement with those obtained from the traditional method, indicating a high level of concordance between the two approaches.

Beyond diagnostic agreement, the mobile-based format demonstrated advantages in scalability, accessibility, and data automation, making it a practical tool for mass deployment in school settings. Students reported ease of use, and supervisors noted reduced time required per screening session compared to manual methods.

### Comparative analysis between CVD and non-CVD students

An independent samples t-test was conducted to compare students’ overall satisfaction with the mobile application between those with and without CVD in Table 2.

**Table 2:** Analysis of t-test results between two groups.

Group Statistics								
	CVD	N	Mean	Std. Deviation	t	df	p	95% CI
OS	Non-CVD	510	3.5917	0.45904				
	CVD	17	3.7065	0.38889	−1.019	525	0.309	[−0.336, 0.107]

As presented in Table 2, students with CVD tended to report slightly higher overall satisfaction with the mobile application than students without CVD. However, the independent-samples t-test indicated that this difference was not statistically significant.

Table 3 presents the results of the independent-samples t-test, confirming that no statistically significant difference was observed between students with and without CVD in their overall satisfaction with the mobile application (p > 0.05).

**Table 3:** Group comparison by color vision deficiency status.

Independent Samples Test										
		Levene’s Test for Equality of Variances		t-test for Equality of Means						
		F	Sig.	t	df	Sig. (2-tailed)	Mean Difference	Std. Error Difference	95% Confidence Interval of the Difference	
									Lower	Upper
OS	Equal variances assumed	2.609	0.107	−1.019	525	0.309	−0.11480	0.11269	−0.33618	0.10657
	Equal variances not assumed			−1.190	17.520	0.250	−0.11480	0.09649	−0.31791	0.08830

### Construct validation for the educational and career counseling application

This section presents the construct validation results for the evaluation instrument designed to measure students’ perceptions of the mobile-based educational and career counseling application. The instrument consists of 18 items categorized into six latent dimensions: awareness (AW), trust in AI (TR), perceived benefits (BE), Barriers and security (BR), system expectations (EX), and willingness to use (RD). To assess the internal consistency and factorial structure of the scale, Cronbach’s alpha reliability analysis was conducted.

As shown in Table 4, the internal consistency of the 18-item educational and career counseling evaluation scale was examined using Cronbach’s alpha. The analysis yielded a Cronbach’s alpha coefficient of 0.857, indicating good reliability. Exploratory factor analysis using principal component analysis with Promax rotation confirmed a six-factor structure, with all items exhibiting strong loadings (> 0.70) on their respective components.

**Table 4:** Reliability analysis of the mobile-based educational and career counseling evaluation scale.

Cronbach’s alpha	Cronbach’s Alpha Based on Standardized Items	N of Items
0.857	0.857	18

To assess the suitability of the data for factor analysis, the Kaiser-Meyer-Olkin (KMO) measure and Bartlett’s Test of Sphericity were conducted, results are presented in Table 5. The KMO value was 0.826, indicating meritorious sampling adequacy (Kaiser, 1974), which suggests that the correlations among items are sufficiently large for factor analysis.

**Table 5:** KMO and Bartlett’s Test of the mobile-based educational and career counseling evaluation scale.

Kaiser-Meyer-Olkin Measure of Sampling Adequacy.		0.826
Bartlett’s Test of Sphericity	Approx. Chi-Square	3601.153
	df	153
	Sig.	0.000

Bartlett’s Test of Sphericity yielded a Chi-square value of 3601.153, with df = 153, and a significance level of p < .001, confirming that the correlation matrix is not an identity matrix and factor analysis is appropriate.

**Fig 3:**
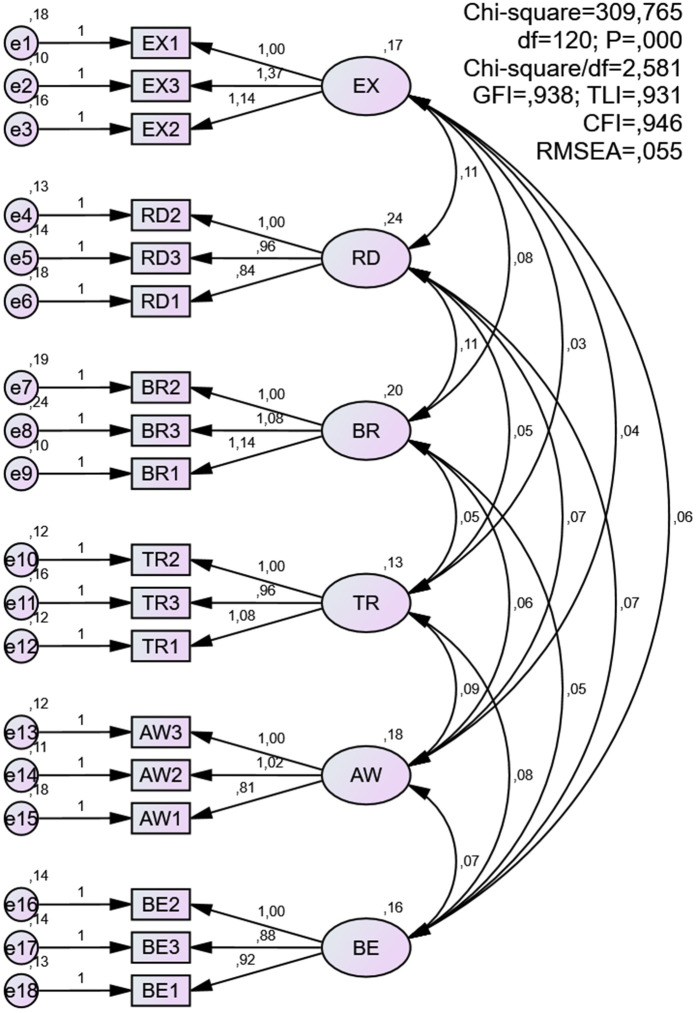
CFA Measurement Model for AI-based Career Counseling.

The Total Variance Explained Table 6 shows that six components have eigenvalues greater than 1, which is the standard threshold for factor retention based on Kaiser’s criterion. Component 1 has an eigenvalue of 5.284, explaining 29.36% of the total variance. The next five components explain 12.91%, 8.69%, 7.10%, 6.78%, and 6.12% of the variance, respectively. Together, the six factors explain a cumulative total of 70.96% of the total variance, indicating a strong representation of the underlying data structure. Exploratory factor analysis revealed a six-factor structure. The result supports the multidimensional nature of the instrument in evaluating students’ perceptions of AI-driven career counseling applications. Confirmatory factor analysis results for the educational and career counseling scale are shown in Fig 3.

**Table 6:** Total variance explained for the mobile-based educational and career counseling evaluation scale.

Component	Initial Eigenvalues			Extraction Sums of Squared Loadings			Rotation Sums of Squared Loadings^a^
	Total	% of Variance	Cumulative %	Total	% of Variance	Cumulative %	Total
1	5.284	29.356	29.356	5.284	29.356	29.356	3.139
2	2.324	12.911	42.267	2.324	12.911	42.267	3.372
3	1.563	8.686	50.953	1.563	8.686	50.953	3.119
4	1.279	7.104	58.056	1.279	7.104	58.056	2.977
5	1.219	6.775	64.831	1.219	6.775	64.831	3.034
6	1.102	6.123	70.955	1.102	6.123	70.955	2.971
7	0.691	3.841	74.795				
8	0.589	3.273	78.068				
9	0.524	2.911	80.979				
10	0.512	2.845	83.824				
11	0.458	2.546	86.370				
12	0.428	2.376	88.746				
13	0.417	2.318	91.064				
14	0.388	2.156	93.220				
15	0.352	1.954	95.174				
16	0.313	1.741	96.915				
17	0.284	1.579	98.494				
18	0.271	1.506	100.000				

Extraction Method: Principal Component Analysis.

^a^When components are correlated, sums of squared loadings cannot be added to obtain a total variance.

The confirmatory factor analysis demonstrated an acceptable model fit, with indices meeting recommended thresholds: χ²(120) = 309.765, p < .001; χ²/df = 2.581; GFI = 0.938; TLI = 0.931; CFI = 0.946; and RMSEA = 0.055. These results provide empirical support for the six-factor structure of the instrument assessing students’ perceptions of the AI-based career counseling application.

Detailed model fit indices from the confirmatory factor analysis are presented in Table 7. The CMIN/DF ratio (2.581) falls within the recommended range of 1–3, indicating a well-fitting model. The Comparative Fit Index (CFI) value of 0.946 is close to the commonly accepted threshold of 0.95, suggesting an acceptable fit. The Standardized Root Mean Square Residual (SRMR) and Root Mean Square Error of Approximation (RMSEA) values are 0.044 and 0.055, respectively, both within excellent ranges. Moreover, the PClose value of 0.140 (> 0.05) suggests that the hypothesis of close fit is not rejected. These results confirm that the proposed six-factor structure provides a statistically sound and conceptually meaningful representation of the observed data.

**Table 7:** Model fit indices for the educational and career counseling evaluation scale.

Measure	Estimate	Threshold	Interpretation
CMIN	309.765	--	--
DF	120	--	--
CMIN/DF	2.581	Between 1 and 3	Excellent
CFI	0.946	>0.95	Acceptable
SRMR	0.044	<0.08	Excellent
RMSEA	0.055	<0.06	Excellent
PClose	0.140	>0.05	Excellent

### Construct validation for the CVD screening application

As shown in Table 8, the mobile-based CVD screening application evaluation scale demonstrated good internal consistency, with a Cronbach’s alpha coefficient of 0.839 across 15 items. This indicates that the scale items reliably capture students’ perceptions across five key dimensions: overall satisfaction, perceived quality and accuracy, time and convenience, enjoyment and user experience, and comparison with traditional methods.

**Table 8:** Reliability analysis of the mobile-based CVD application evaluation scale.

Cronbach’s Alpha	Cronbach’s Alpha Based on Standardized Items	N of Items
0.839	0.841	15

As shown in Table 9, the Kaiser-Meyer-Olkin measure indicated adequate sampling adequacy (KMO = 0.804), and Bartlett’s Test of Sphericity was statistically significant (p < 0.001), supporting the suitability of the data for factor analysis. Together, these results provide strong justification for proceeding with factor extraction to explore the underlying dimensions of user perceptions related to the mobile-based CVD screening tool.

**Table 9:** KMO and Bartlett’s Test of the mobile-based CVD application evaluation scale.

KMO and Bartlett’s Test		
Kaiser-Meyer-Olkin Measure of Sampling Adequacy.		0.804
Bartlett’s Test of Sphericity	Approx. Chi-Square	3105.972
	df	105
	Sig.	0.000

**Fig 4:**
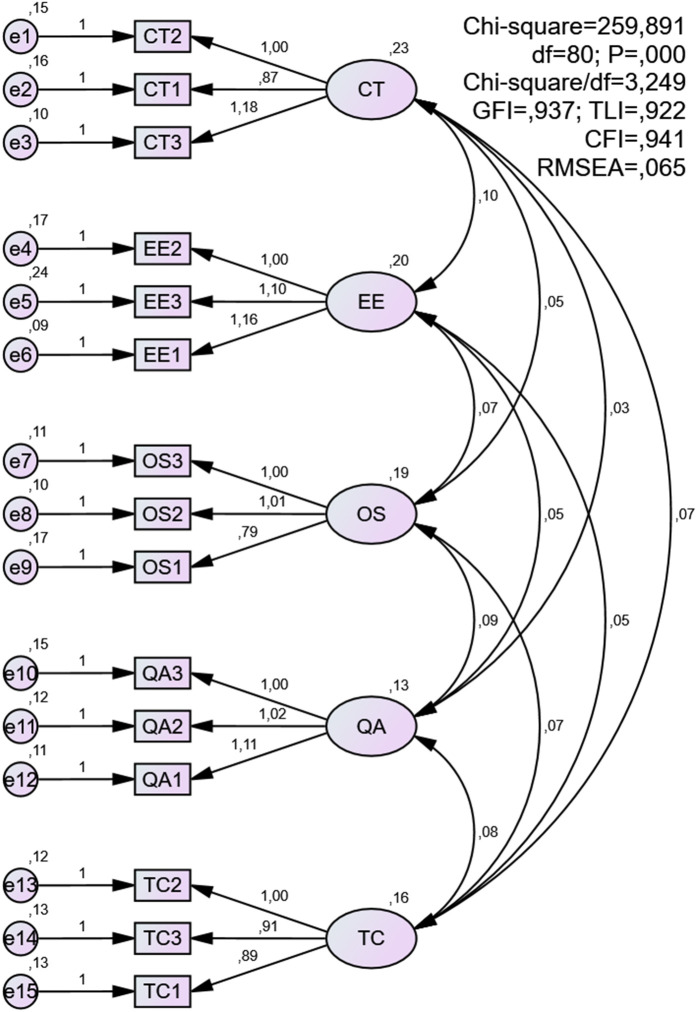
CFA Measurement Model of the mobile-based CVD application evaluation scale.

The exploratory factor analysis (Table 10) of the mobile-based CVD application evaluation scale extracted five components with eigenvalues greater than 1, explaining a cumulative 72.17% of the total variance. The first factor accounted for 31.11%, followed by subsequent components explaining 14.28%, 10.43%, 8.61%, and 7.74%, respectively. This level of explained variance exceeds the standard threshold of 60%, indicating that the factor structure effectively captures the underlying constructs. The results confirm the multidimensionality of the instrument and provide strong evidence for its construct validity in evaluating user experience with the CVD screening application. Confirmatory factor analysis results for the CVD screening application evaluation scale are shown in Fig 4.

**Table 10:** Total Variance Explained of the mobile-based CVD application evaluation scale.

Total Variance Explained							
Component	Initial Eigenvalues			Extraction Sums of Squared Loadings			Rotation Sums of Squared Loadings^a^
	Total	% of Variance	Cumulative %	Total	% of Variance	Cumulative %	Total
1	4.666	31.107	31.107	4.666	31.107	31.107	2.820
2	2.143	14.284	45.390	2.143	14.284	45.390	2.870
3	1.565	10.432	55.823	1.565	10.432	55.823	2.995
4	1.291	8.605	64.427	1.291	8.605	64.427	3.023
5	1.161	7.739	72.166	1.161	7.739	72.166	2.845
6	0.644	4.295	76.462				
7	0.556	3.705	80.167				
8	0.509	3.396	83.563				
9	0.451	3.006	86.568				
10	0.417	2.781	89.349				
11	0.394	2.626	91.975				
12	0.334	2.227	94.202				
13	0.315	2.103	96.305				
14	0.296	1.972	98.276				
15	0.259	1.724	100.000				

Extraction Method: Principal Component Analysis.

^a^When components are correlated, sums of squared loadings cannot be added to obtain a total variance.

All standardized factor loadings for the 15 observed items were above the recommended threshold of 0.70, suggesting that each item meaningfully contributes to its corresponding latent construct. The five dimensions-Overall Satisfaction (OS), Quality and Accuracy (QA), Time and Convenience (TC), Enjoyment and User Experience (EE), and Comparison with Traditional Methods (CT)-were distinctly identified and reliably measured. These findings confirm the construct validity of the instrument and its appropriateness for assessing students’ perceptions of the mobile-based CVD screening tool.

The CFA (Table 11) provided evidence supporting the validity of the five-factor structure of the CVD application evaluation instrument. The model exhibited an acceptable fit across key goodness-of-fit indicators: Chi-square (χ²) = 259.891, degrees of freedom (df) = 80, p < .001; the ratio χ²/df = 3.249 was within the acceptable range (< 5). Additional fit indices also met recommended thresholds, including GFI = 0.937, TLI = 0.922, CFI = 0.941, and RMSEA = 0.065. These values indicate that the proposed model adequately represents the observed data.

**Table 11:** Model fit indices for the mobile-based CVD application evaluation scale.

Measure	Estimate	Threshold	Interpretation
CMIN	259.891	--	--
DF	80	--	--
CMIN/DF	3.249	Between 1 and 3	Acceptable
CFI	0.941	>0.95	Acceptable
SRMR	0.049	<0.08	Excellent
RMSEA	0.065	<0.06	Acceptable
PClose	0.002	>0.05	Below recommended threshold

### Comparative analysis of the AI-based application and traditional screening methods

The mobile-based color vision screening application enables rapid, accurate, and user-friendly assessment among high school students. The initial version was built on the standard 38 Ishihara plates, incorporating randomized presentation, controlled display time, and automated recording of individual outcomes. The system was piloted on Android smartphones, with an average screening duration of 3–5 minutes per student, notably shorter than the conventional paper-based Ishihara test.

The application was optimized for real-time processing, immediate result generation, and secure storage of anonymized screening data. Across all pilot sessions, the system operated reliably, with no software failures or interruptions observed. A minimalist interface with neutral background tones was adopted to ensure adequate luminance contrast and accessibility for students with CVD.

During the initial pilot phase (n = 20), the application recorded only overall screening outcomes to assess system stability and user interaction. Based on these results, the data acquisition algorithm was refined to enable plate-level response logging. The upgraded version was subsequently tested in a larger sample (100 students), allowing detailed identification of misrecognized plates and supporting classification of CVD subtypes. Comparison with the printed Ishihara test showed complete agreement across tested students, confirming the reliability of the mobile-based screening tool.

The results in Table 12 show that, after pilot testing with 100 students, the application was deployed at scale with 527 students from five high schools. All 17 students identified by the application as having CVD were re-evaluated using standard printed Ishihara tests, showing complete agreement across all classifications and confirming the accuracy of the application for preliminary school-based screening.

**Table 12:** Comparison between traditional method and AI-assisted screening application*.

*Indicator*	*Paper-based Ishihara test*	*Mobile application*
Total number of students	100	100
Red–green CVD	2	2
Deutan	2	2
Protan	0	0
Achromatopsia	1	1
Total number of students with CVD	5	5

* Results are based on the validation subsample of 100 students.

The software required only 3–5 minutes per student, compared with 10–15 minutes for the paper-based method, and allowed simultaneous screening of multiple students. In addition, the AI-assisted system provides personalized educational and career recommendations following screening, extending beyond diagnosis to individualized educational support. Student satisfaction with time efficiency and convenience is shown in Table 13.

**Table 13:** One-sample t-test results for time efficiency and convenience.

One-Sample Test						
	Test Value = 0					
	t	df	Sig. (2-tailed)	Mean Difference	95% Confidence Interval of the Difference	
					Lower	Upper
TC1	158.764	526	0.000	3.512	3.47	3.56
TC2	151.414	526	0.000	3.535	3.49	3.58
TC3	155.083	526	0.000	3.507	3.46	3.55

The one-sample t-test results showed that students’ ratings for time efficiency and convenience were significantly higher than the neutral midpoint (p < 0.001). Mean scores ranged from 3.51 to 3.54 on the five-point scale, indicating favorable perceptions of the application in terms of time savings, flexibility, and ease of use. The 95% confidence intervals were entirely above the neutral value, confirming the robustness of the findings.

Students’ satisfaction with the mobile application compared with the paper-based method was assessed using a continuous five-point scale. The reliability analysis showed that the three-item scale measuring comparison with traditional methods (CT1-CT3) demonstrated good internal consistency, with Cronbach’s alpha values ranging from 0.725 to 0.806, indicating that the scale was suitable for further analysis (Table 14).

**Table 14:** Reliability of the comparison with the traditional method scale.

Item-Total Statistics					
	Scale Mean if Item Deleted	Scale Variance if Item Deleted	Corrected Item-Total Correlation	Squared Multiple Correlation	Cronbach’s Alpha if Item Deleted
CT1	7.22	1.314	0.649	0.424	0.806
CT2	7.30	1.209	0.695	0.494	0.761
CT3	7.26	1.109	0.731	0.537	0.725

Results of the one-sample t-test revealed that all comparison criteria had significantly positive mean differences (p < 0.001), with 95% confidence intervals entirely above the neutral value. These findings indicate that students consistently rated the mobile application more favorably than the traditional paper-based test in terms of preference, clarity in understanding color vision status, and overall convenience (Table 15).

**Table 15:** One-sample test results for the comparison with the traditional method.

One-Sample Test						
	Test Value = 0					
	t	df	Sig. (2-tailed)	Mean Difference	95% Confidence Interval of the Difference	
					Lower	Upper
CT1	145.415	526	0.000	3.670	3.62	3.72
CT2	135.198	526	0.000	3.590	3.54	3.64
CT3	129.497	526	0.000	3.626	3.57	3.68

Overall, these findings indicate that the proposed mobile-based color vision screening tool is accurate, stable, and user-friendly, with strong potential for large-scale implementation and future integration with AI-driven educational and career counseling modules in secondary school settings.

## Discussion

### Key findings and implications

This study confirms the feasibility and effectiveness of integrating CVD screening with AI-based educational counseling through a mobile application, specifically designed for high school students in Viet Nam. Compared to the traditional Ishihara paper test, the digital platform achieved comparable diagnostic accuracy while offering notable advantages in time efficiency, suitability for mass deployment, and the provision of personalized guidance.

The prevalence of CVD observed among male students in the present study is consistent with previous epidemiological evidence indicating that congenital CVD occurs predominantly in males because of its X-linked inheritance pattern [1,2,4]. This finding highlights persistent gender disparities in color vision health. Importantly, the application accurately distinguished among different types of CVD (red-green, protan, deutan, and achromatopsia), demonstrating reliable screening performance and a high level of agreement with the conventional Ishihara test. These results indicate that AI-assisted screening technologies can be meaningfully applied in large-scale educational contexts.

### Theoretical and practical contributions

This research contributes to the growing intersection of AI and education in several key ways. First, it introduces a novel integration of health screening and educational counseling-two domains traditionally addressed separately in schools. Second, it validates the use of AI for providing tailored counseling to students with visual impairments, an area that has been largely overlooked in prior studies. Third, it demonstrates the value of mobile-based platforms in promoting equitable access to health and guidance services in resource-limited educational settings.

The study also provides strong psychometric support for the evaluation instruments employed, with Cronbach’s alpha coefficients of 0.857 and 0.839, and KMO values above 0.80. These metrics affirm the internal consistency and construct validity of the scales used to measure students’ perceptions and experiences with the digital tools.

The absence of CVD cases among female students in this sample is consistent with the X-linked inheritance pattern of congenital CVD, though larger samples may be needed to detect rare female cases.

### Challenges and development opportunities

Despite its potential, the application of AI in educational counseling still faces challenges. Technical limitations, data management, and concerns around privacy and ethical use of student information remain pressing issues. Furthermore, ensuring algorithmic fairness and maintaining the accuracy and transparency of AI-generated recommendations requires ongoing development, testing, and refinement.

Nevertheless, this also represents a significant opportunity to modernize traditional counseling approaches, improve personalization, and scale support to reach more students. Future system upgrades may include advanced machine learning, big data analytics, and improved user interface designs, which will strengthen the system’s adaptability and acceptance.

### Practical recommendations for research and implementation

To enhance the utility and scalability of AI-assisted educational counseling tools, the following recommendations are proposed:

- Develop inclusive and adaptable versions of the application that can accommodate diverse technical and geographical contexts, ensuring nationwide accessibility, especially in underserved and rural areas.
- Provide training for school counselors and educators to effectively adopt and integrate AI tools into educational and career guidance practices.
- Expand the functionality of the application to include assessments of other personal factors such as soft skills, career interests, and additional learning difficulties or disabilities.
- Establish stringent data protection and privacy protocols, ensuring the ethical use of student information in compliance with national and international standards.
- Conduct future studies with larger and more diverse samples, incorporating both quantitative and qualitative methods to comprehensively evaluate the effectiveness and usability of AI in educational guidance.

These recommendations aim to support the sustainable integration of AI technology into educational ecosystems and promote equity in career counseling services for all students.

## Conclusion

This experimental study has demonstrated the effectiveness and feasibility of applying an AI-based mobile application for CVD screening and educational counseling among high school students in Danang, Viet Nam. The software not only enabled accurate and timely detection of CVD but also provided personalized counseling recommendations, thereby enhancing students’ awareness, trust, and satisfaction with the career guidance process.

The findings contribute to a growing body of evidence supporting the role of AI in innovating educational counseling methods, particularly in improving accessibility and personalized support within secondary education systems.

Future research should focus on developing contextually adaptable software versions that address technical, cultural, and geographical differences, while also incorporating more comprehensive assessments of students’ competencies, interests, and individual needs. Further studies on data security protocols and implementation strategies in diverse educational environments are also necessary to ensure the long-term sustainability and scalability of such systems. Expanding the sample size and diversity in future evaluations will help strengthen empirical evidence and validate the practical utility of AI-based counseling solutions in education.

## Supporting information

S1 File De-identified minimal dataset underlying the findings reported in this study. This file contains the anonymized survey dataset used for the statistical analyses reported in the manuscript.
(XLSX)

S2 File Student survey questionnaire on AI-based educational and career counseling. This file contains the English version of the questionnaire used to collect students’ responses in the study.
(PDF)
