# Supplementary material for: AI-based color vision screening and educational counseling for high school students: An experimental study
Source: PLoS One. 2026 Jul 22;21(7):e0353871. doi: 10.1371/journal.pone.0353871 (PMC13390841; doi:10.1371/journal.pone.0353871)
Supplement: S2 File — This file contains the English version of the questionnaire used to collect students’ responses in the study. (PDF) [file pone.0353871.s002.pdf]

Appendix 2

STUDENT SURVEY QUESTIONNAIRE ON THE ROLE OF AI IN EDUCATIONAL AND CAREER COUNSELING

Dear students, We are conducting a study on the application of Information Technology in assessing color vision deficiency to support educational counseling for high school students in Danang. We hope to receive your opinions by asking you to answer the following questions. Your responses will help improve the quality of this research. We also commit to keeping your responses, as well as your personal information, confidential. All collected results will be used solely for research purposes and not for any other purpose.

We sincerely thank you for your valuable contribution!

Instructions:

After installing the mobile application for color vision deficiency screening and educational/career counseling, you will answer the questions to identify your color vision status. You will then proceed to receive counseling on academic majors and career fields based on your interests, or click on the available groups of academic majors in the system to receive counseling.

At the same time, please help us complete this additional survey by marking "✓" in the box that best reflects your opinion.

5 = highest level

1 = lowest level

Each of your responses contributes to the completion of this research. Thank you very much!

Part 1. General Information

- Gender: Male / Female / Other
- Grade: 10 / 11 / 12
- School: ______________________________

Part 2. Evaluation of the Mobile Educational and Career Counseling Application

Please indicate your level of agreement with the following statements using a scale from 1 to 5 (1 = Strongly disagree; 5 = Strongly agree).

Standard	Criteria/Indicators	1	2	3	4	5
**1. Awareness of career counseling (AW)**	Level of participation in career counseling activities at school					
	The role of career counseling in choosing future academic majors and careers					
	The impact of the contents provided in the career counseling session, such as personal abilities, career information, soft skills, etc.					
**2. Trust in AI counseling (TR)**	The AI system can provide appropriate career counseling based on users' abilities					
	The AI system can provide suitable choices based on personal interests					
	The AI system is effective when integrated with color vision test results					
**3. Perceived benefits (BE)**	AI helps save time and optimize the counseling process					
	AI helps personalize career recommendations for each student					
	AI provides good support for students with special needs, such as color blindness, learning disorders, etc.					
**4. Barriers and security (BR)**	The AI system will ensure the confidentiality of personal information					
	The system is designed to be easy to use in career counseling					
	Counseling results generated by the AI system are reliable and accurate					
**5. Expectations of the**	The counseling system has a simple and student-friendly interface					

system (EX)	The counseling system can support online interaction in choosing academic majors and careers					
	The counseling system effectively meets the needs of students with color vision deficiency					
**6. Willingness to Use (RD)**	I am willing to use the AI-integrated career counseling system if it is implemented at my school					
	I am willing to recommend the AI counseling system to my friends					
	I am willing to participate in training sessions to use this AI counseling system					

Part 3. Evaluation of the Mobile Color Vision Screening Application

Please indicate your level of agreement with the following statements using a scale from 1 to 5 (1 = Strongly disagree; 5 = Strongly agree).

Standard	Criteria/Indicators	1	2	3	4	5
**1. Overall satisfaction with the application (OS)**	I am satisfied with the interface of the mobile color vision screening application					
	The application is easy to use and operate					
	I feel that the application responds quickly and runs smoothly					
**2. Quality and accuracy of the screening (QA)**	The color vision test results provided by the application are accurate and reliable					
	I trust that the application can correctly detect my color vision status					
	The application provides clear and understandable information and explanations about the test results					
**3. Time and convenience in taking the test (TC)**	The time required to complete the test on the application is appropriate and not boring					
	Taking the test on the application is faster than using the traditional paper-based chart					
	I can complete the test anytime and anywhere using the application					
**4. Interest and user experience (EE)**	I feel interested when using the mobile color vision screening application					
	The application makes color vision testing more interesting than the traditional method					
	I would like to recommend this application to my friends or relatives					
**5. Comparison with the traditional method (CT)**	Compared with the paper-based color vision test chart, I prefer using the application					
	The application helps me better understand my color vision status compared with the traditional method					
	I find the application more accessible and convenient than taking the test on paper					

Other comments: ....

Thank you very much for your cooperation!

==============================
